# Cost-Effectiveness of Different Cervical Screening Strategies in Islamic Republic of Iran: A Middle-Income Country with a Low Incidence Rate of Cervical Cancer

**DOI:** 10.1371/journal.pone.0156705

**Published:** 2016-06-08

**Authors:** Azin Nahvijou, Rajabali Daroudi, Mamak Tahmasebi, Farnaz Amouzegar Hashemi, Mohsen Rezaei Hemami, Ali Akbari Sari, Ahmad Barati Marenani, Kazem Zendehdel

**Affiliations:** 1 Cancer Research Center, Cancer Institute of Iran, Tehran University of Medical Sciences, Tehran, I. R. Iran; 2 Department of Health Management and Economics, School of Public Health, Tehran University of Medical Sciences, Tehran, I. R. Iran; 3 Institute of Health & Wellbeing Health Economics & Health Technology Assessment University of Glasgow, Scotland, United Kingdom; 4 Department of Health Services Management, School of Health Management and Information Sciences, Iran University of Medical Sciences, Tehran, I. R. Iran; 5 Cancer Model Research Center, Cancer Institute of Iran, Tehran University of Medical Sciences, Tehran, I. R. Iran; Bharathidasan University, INDIA

## Abstract

**Objective:**

Invasive cervical cancer (ICC) is the fourth most common cancer among women worldwide. Cervical screening programs have reduced the incidence and mortality rates of ICC. We studied the cost-effectiveness of different cervical screening strategies in the Islamic Republic of Iran, a Muslim country with a low incidence rate of ICC.

**Methods:**

We constructed an 11-state Markov model, in which the parameters included regression and progression probabilities, test characteristics, costs, and utilities; these were extracted from primary data and the literature. Our strategies included Pap smear screening and human papillomavirus (HPV) DNA testing plus Pap smear triaging with different starting ages and screening intervals. Model outcomes included lifetime costs, life years gained, quality-adjusted life years (QALY), and incremental cost-effectiveness ratios (ICERs). One-way sensitivity analysis was performed to examine the stability of the results.

**Results:**

We found that the prevented mortalities for the 11 strategies compared with no screening varied from 26% to 64%. The most cost-effective strategy was HPV screening, starting at age 35 years and repeated every 10 years. The ICER of this strategy was $8,875 per QALY compared with no screening. We found that screening at 5-year intervals was also cost-effective based on GDP per capita in Iran.

**Conclusion:**

We recommend organized cervical screening with HPV DNA testing for women in Iran, beginning at age 35 and repeated every 10 or 5 years. The results of this study could be generalized to other countries with low incidence rates of cervical cancer.

## Introduction

Invasive cervical cancer (ICC) is the fourth most common cancer among women worldwide [1]. The highest age-standardized incidence rate (ASR) has been reported in Eastern Africa (ASR: 42.7 per 100,000) in 2013) [1]. ICC is a preventable disease, and cervical screening programs using the Pap smear procedure have an essential role in reducing the incidence and mortality rate of ICC in many countries, including the United States, United Kingdom, and Nordic countries, among others [2–4]. This decline seems to be limited to high-income countries, but 80% of ICCs occur in low- and middle-income countries [5, 6].

Human papillomavirus (HPV) infection is known as a necessary cause of ICC [7, 8]. As a result, HPV DNA testing has recently emerged as a novel and effective screening method. HPV vaccination has been widely recommended for prevention of HPV infection and therefore, prevention of ICC [9]. HPV DNA testing methods have higher sensitivity than Pap smear testing for detection of precancerous lesions [10]; therefore, Pap smear screening requires more frequent testing with shorter intervals compared with HPV DNA testing. Another limitation of Pap smear is its dependency on cytological techniques, which require relatively more highly trained personnel compared with HPV DNA testing, in which advanced and automated are used [11]. Moreover, aside from the type of screening test, the starting age, screening intervals, and participation rate of women are important factors for an effective screening program. For both Pap smear and HPV testing methods, specificity is likely to increase with increased age at screening [12], and HPV triage of women aged 35 or older is more effective than in younger women [13, 14].

Cost is another characteristic of a screening program that should be taken into account along with its effectiveness. In fact, health policy makers tend to choose the most cost-effective screening method for public health programs. Results of economic evaluation studies may be important in such decision-making processes [15]. Cost-effectiveness studies provide an analytical tool to decide which intervention is suitable for implementation of screening in the target population [16]. However, most studies comparing Pap smear with HPV DNA testing for cervical screening have been conducted in developed countries and data from low-and middle-income countries are limited [17].

The incidence rate of ICC is about 5 per 100,000 in the Islamic Republic of Iran, and its peak is a decade earlier than that in high-income countries [18]. Because of the low incidence rate of ICC, there is no organized program for prevention of ICC in Iran, and patients are usually diagnosed in advanced stages with poor prognosis. Women who are 21–69 years of age are offered Pap smear testing every 3 years if they are referred to health care centers or a gynecologist for any reason. In this approach, called opportunistic screening, the decision about screening take place in the clinical setting and depends on the initiative of each woman or her doctor [19]. In opportunistic screening, some women may be screened frequently whereas others may never attend the screening. This type of screening is ineffective and has been discouraged because of its poor quality and low coverage [19, 20]. In contrast, an organized screening program is a high standard of service that offers screening to the target population by active invitation and follow-up. An organized program is usually monitored regularly to ensure the program’s coverage and quality. Based on a recent priority setting study, development and implementation of an organized screening program and decisions about the proper age, intervals, and tests for screening are considered important priorities for Iran [21].

In this study, we aimed to perform an economic evaluation and suggest the most cost-effective ICC screening strategy, including the type of test and screening schedule for low-risk populations in Iran. The results of this study could be generalized to other countries with low incidence rates of ICC in which governments are hesitant to introduce cervical screening programs.

## Materials and Methods

### Decision Model

We constructed an 11-state Markov cohort model of women to estimate the lifetime cost, quality-adjusted life years (QALY), incidence number, and mortality of ICC using 11 different screening strategies in comparison to no screening strategy. The time horizon was lifetime. All costs and QALY were discounted at an annual rate of 3%. All analyses were performed using TreeAge Pro 2011 software (TreeAge Software Inc., Williamstown, MA, USA).

### Natural History of ICC

We modeled the natural history of ICC in 11 health states, including healthy, HPV infection, low-grade squamous intraepithelial lesion (LSIL), high-grade squamous intraepithelial lesion (HSIL), cancer stage I, cancer stage II, cancer stage III, cancer stage IV, death from cancer, death from other causes, and survived state. We simulated a cohort in which a healthy woman can move between different states in the Markov model. Each cycle was equal to 1 year, and the model simulated the natural history of ICC in women over 15 years of age.

During simulation in the healthy state, women could remain healthy or progress to the HPV infection state in one cycle. Women with HPV infection could progress to the LSIL or HSIL state, stay in the HPV infection state, or regress to the healthy state in 1 year. Every woman in the LSIL state could remain in the LSIL state, progress to the HSIL state, or regress to the HPV infection or healthy state. Women in the HSIL state could progress to cancer stage I, remain in the same state, or regress to the LSIL, HPV infection, or healthy state. Women in each of the four stages of cancer (I–IV) could only progress to the next stage of cancer, remain at the same stage, or die of cancer. We considered no regression for ICC in the model. If a woman survived for 5 years, she could progress to the survived state. Each woman in any state could die from other causes.

### Model Assumptions

Our model had the following assumptions:

1. All women undergo the screening program, and the screening coverage is 100%.
2. Every woman entered into the model is in the healthy state.
3. Even after clearance from HPV infection and treatment, there is a chance of reinfection with HPV.
4. Every woman with abnormal cervical cells will return to a routine screening program immediately after follow-up and treatment of her precancerous lesions.

### Screening Strategies

We conducted a cost-effectiveness study and compared 11 screening strategies with no screening in terms of costs and effectiveness. One strategy was the current screening recommendation in Iran, i.e., cervical screening every 3 years for women ages 21–69 years. Other strategies were selected based on comparing new screening technology (HPV DNA testing) and old technology (Pap smear) with a combination of different starting ages and screening intervals. The starting age ranged from 21 to 35 years and intervals were 3, 5, and 10 years. Pap smear triage was used for all strategies.

We stopped all strategies in our model at the age of 65 years and the simulation model continued until all women in the cohort had died. We used international guidelines for the process of each screening method (i.e., Pap smear or HPV DNA testing) and then consulted with gynecologists so as to standardize the process for Iran. We have published details about the process elsewhere [22].

We did not include visual inspection with 4% acetic acid (VIA) because it has the lowest sensitivity and specificity and is usually recommended for low-resource settings [23–25]. In addition, we did not compare liquid-based cytology (LBC) in this analysis because there are no significant differences between LBC and Pap smear in terms of test characteristics and LBC has a higher cost compared with conventional Pap smear [26].

### Description of Strategies

Pap smear 21–3: Pap smear starting at age 21 for 3 consecutive years and then every 3 years.

Pap smear 30–3: Pap smear starting at age 30 with 3-year intervals.

Pap smear 30–5: Pap smear starting at age 30 with 5-year intervals.

Pap smear 30–10: Pap smear starting at age 30 with 10-year intervals.

Pap smear 35–3: Pap smear starting at age 35 with 3-year intervals.

Pap smear 35–5: Pap smear starting at age 35 with 5-year intervals.

Pap smear 35–10: Pap smear starting at age 35 with 10-year intervals.

HPV 30–5: HPV DNA testing starting at age 30 with 5-year intervals.

HPV 30–10: HPV DNA testing starting at age 30 with 10-year intervals.

HPV 35–5: HPV DNA testing starting at age 35 with 5-year intervals.

HPV 35–10: HPV DNA testing starting at age 35 with 10-year intervals.

### Model Parameters

#### Parameters of the natural history

We found no data of the incidence rate of HPV infection in Iran. Therefore, similar to previous studies [27–32], we used calibration methods based on the stage and age-specific rate of ICC in Iran to estimate the age-specific incidence rate of HPV infection, using data from the national cancer registry and Globocan 2012 [1, 33]. Because the ranges of progression and regression of HPV incidence are fairly consistent internationally, we used data from the literature to obtain the regression, progression, and persistent probability for each state [28,29,34,35].

We obtained the mortality rate of the general Iranian population from the life table published by the World Health Organization (WHO) for Iran [36]. In addition, we used 1-, 2-, 3-, 4-, and 5-year survival of ICC and stage-specific survivals based on a recent multicenter study in the capital city of Tehran [37] (Table 1).

**Table 1:** Model parameters (transition probabilities, screening test characteristics, quality of life, and costs), baseline values, and references.

Parameters	Base case	References
Natural History		
Probability of transition of Well to HPV	Age-dependent	calibration
Probability of transition		[45,47–49]
Probability of Transition of HPV to Well 15–85 years	0.7	
Probability of Transition of HPV to LSIL 15–85 years	0.072	
Probability of Transition of HPV to HSIL		
15 years	0.032	
85 years	0.042	
Probability of Transition of LSIL to Well		
15 years	0.16	
85 years	0.081	
Probability of Transition of LSIL to HPV		
15 years	0.16	
85 years	0.082	
Probability of Transition of LSIL to HSIL		
15 years	0.017	
85 years	0.069	
Probability of Transition of HSIL to Well	0.069	
Probability of Transition of HSIL to HPV 15–85 years	0.05	
Probability of Transition of HSIL to LSIL 15–85 years	0.069	
Probability of Transition of HSIL to cancer I		
15 years	0.01	
85 years	0.005	
Probability of detection cancer		[46,49,50]
Probability of detection stage I cancer	0.15	
Probability of detection stage II cancer	0.23	
Probability of detection stage III cancer	0.6	
Probability of detection stage IV cancer	0.9	
Progression Cancer I to stage II cancer	0.437	
Progression Cancer II to stage III cancer	0.535	
Progression Cancer III to stage IV cancer	0.683	
Survival		[67]
Survival stage I cancer	0.86	
Survival stage II cancer	0.63	
Survival stage III cancer	0.35	
Survival stage IV cancer	0.11	
Test Characteristics		
Sensitivity of Pap smear	0.66	[38]
Specificity of Pap smear	0.86	[38]
Sensitivity of HPV DNA testing	0.81	[14,43]
Specificity of HPV DNA testing	095	[14,43]
Specificity of colposcopy	1	[27]
Quality of Life(QALY)		
Quality of life of well state	1	[40,52]
Quality of life of LSIL state	0.97	[40,52]
Quality of life of HSIL state	0.93	[40]
Quality of life of stage I patients	0.85	Observational
Quality of life of stage II patients	0.79	Observational
Quality of life of stage III patients	0.18	Observational
Quality of life of stage IV patients	0.14	Observational
Cost(US$)		
Cost of HPV DNA testing	10	Observational
Cost of Pap screening	4	[22]
Cost of biopsy of cervix	15	[22]
Cost of visit	5	[22]
Cost of colposcopy	13	[22]
Cost of conization	372	[22]
Cost of stage I cancer	2,207	Observational
Cost of stage II cancer	3,449	Observational
Cost of stage III cancer	2,485	Observational
Cost of stage IV cancer	2,864	Observational
Cost of terminal care of stage I patients	1108	Observational
Cost of terminal care of stage II patients	1732	Observational
Cost of terminal care of stage III patients	1248	Observational
Cost of terminal care of stage IV patients	1438	Observational
Cost of follow-up for cancer	497	Observational

#### Characteristics of the screening test

Because HPV DNA testing is not currently used for cervical screening in Iran, we failed to find local HPV DNA test characteristics. We obtained test characteristics for HPV DNA testing using polymerase chain reaction (PCR) from a published systematic review of the literature [14] and from locally published data [38] for the Pap smear test (Table 1).

#### Utility

We used QALY for evaluating the effectiveness of cervical screening, and the validated European Quality of Life (EuroQoL) EQ-5D questionnaire [39] for patients diagnosed with ICC based on their stage. QALY for healthy, HPV, LSIL, and HSIL states were obtained from the international literature [40] (Table 1).

#### Cost data

We used direct medical costs from a health provider perspective. The screening cost included the cost of the test (HPV or Pap), physician visit, colposcopy, biopsy, and conization [22]. In addition, we collected data from three cancer hospitals in Tehran and estimated the treatment costs for different stages of ICC [41]. All costs were measured based on 2013 Iranian tariffs and converted to 2013 USD. Because few laboratories in Iran currently use HPV DNA testing as their diagnostic procedure, we had no available price for this study. We therefore consulted with manufacturers regarding the potential costs for HPV DNA testing for screening purposes in Iran. Estimated costs were then used in this economic analysis. We used the lowest prices provided by manufacturers as the base case and higher prices for one-way sensitivity analysis (Table 1).

### Cost-Effectiveness Analysis

We calculated incremental cost-effectiveness ratios (ICERs) to compare the cost and effectiveness of each screening strategy, and no screening. The strategy with the highest cost and least effectiveness was considered the dominated strategy, and the strategy with the greatest effectiveness and lowest cost the dominant or most cost-effective strategy.

We used the gross domestic product (GDP) per capita, which is suggested by the WHO as the threshold for the most cost-effective strategy [42]. We used 1- and 2-fold GDP per capita as the threshold to compare the selected strategies. We also compared strategies using undiscounted effectiveness and life years gained (LYG) instead of QALY.

### Sensitivity Analysis

We performed a one-way sensitivity analysis to estimate the impact of uncertainty in the different parameters, including test characteristics (sensitivity and specificity), and the costs of HPV DNA testing and Pap smear testing methods. We considered ranges from 71.6–98.7 for sensitivity of HPV DNA testing and ranges from 81.9–96.9 for specificity of HPV DNA testing. For Pap smear, ranges for sensitivity and specificity were 63.9–84.7 and 90.2–97.3, respectively. All data of test characteristics were obtained from the literature [14, 43]. Regarding cost, we used 50%–75% above and below the estimated cost for sensitivity analysis.

## Results

### Model Validation

Our model predictions for age-specific ICC incidence and mortality rates were highly consistent with the available empirical data. The age-specific incidence rate of HPV infection predicted by the model was 0.06 at age 45 years and the age-specific incidence rate of ICC was estimated to be 17.09 per 100,000 among women aged 50–54 years. This result was within a plausible range obtained from the published literature [44]. The mortality to incidence ratio was 0.38, which was consistent with the ratio calculated by Globocan 2012 for Iran. [1].

### Base Case

**Fig 1:**
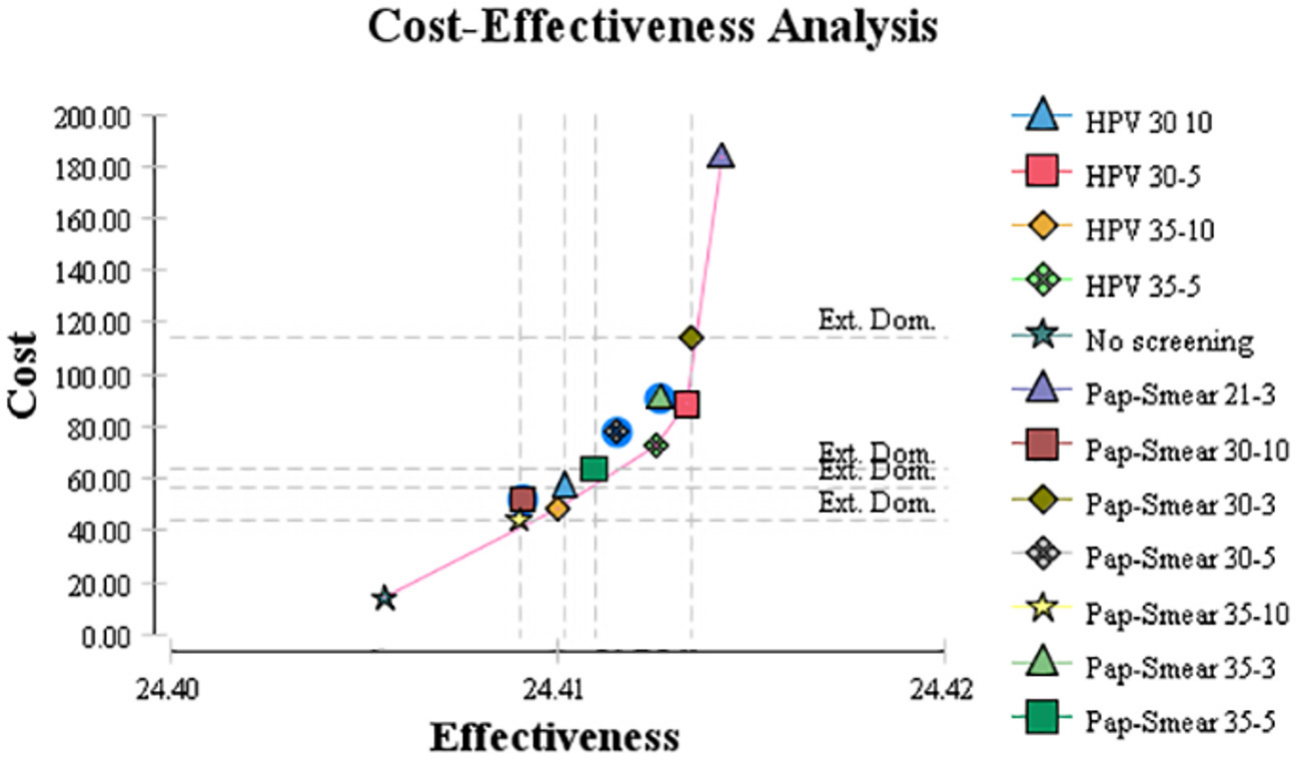
Cost-effectiveness of 11 different strategies for cervical screening. Strategies standing on the curve are dominant strategies, indicating that they cost less and had more effect. Strategies located out of the curve are dominated strategies, i.e. their costs were much higher than their effects.

In the base case analysis, the incidence rates, mortality rates, QALY, costs, and ICERs of 11 screening strategies were compared with no screening (Table 2). The incidence and mortality of ICC decreased from 1,322 and 509 patients in the no-screening strategy to 483 and 184 patients using Pap smear starting from age 21 with 3-year intervals, respectively. Reductions in the incidence and mortality rates of each strategy depended on the screening methods, starting ages, and screening intervals. We found that HPV DNA testing begun at age 35 years and repeated every 10 years with an ICER of $8,875 per QALY was the most cost-effective strategy (Fig 1). ICERs of all strategies were more than 1-fold the GDP per capita. Except for Pap smear begun at age 21 and repeated every 3 years, all other strategies were less than 2-fold the GDP per capita. The GDP per capita for Iran was $6,631 in 2013.

**Table 2:** Incidence, mortality, cost, effectiveness, ICER, and undiscounted ICER of 11 strategies compared with no screening strategy.

Strategies	Incidence(No of cases)	Mortality(No of cases)	Cost($)	QALY	ICER(US $/QALY)	Undiscounted ICER
No screening	1321	509	13.93	24.406	0	0
HPV 35–10	940	363	42.89	24.409	8,875	2,472
Pap smear35-10	591	229	45.39	24.409	9,080	2,589
HPV 35–5	727	281	63.69	24.411	9,087	2,554
Pap smear 35–5	779	300	66.24	24.411	9,650	2,747
HPV 30–5	674	259	76.94	24.412	10,248	2,978
HPV 30 10	933	359	50.20	24.409	10,695	3,069
Pap smear 30–5	731	281	81.03	24.412	11,189	3,276
Pap smear 30–10	974	375	53.75	24.409	11,193	3,261
Pap smear 35–3	611	236	94.70	24.413	11,362	3,251
Pap smear 30–3	543	208	119.30	24.413	13,342	3,920
Pap smear 21–3	483	184	192.04	24.414	20,492	6,234

Although Pap smear was the cost-effective method when using LYG as the outcome, HPV DNA testing became the most cost-effective when undiscounted QALY was considered the outcome measure (Table 3).

**Table 3:** The most cost-effective strategies (CES) based on different calculation methods. The cost-effectiveness of 11 strategies were estimated with different methods. Effectiveness was measured with QALY, LYG, and undiscounted QALY.

Calculation methods of cost-effectiveness	First CES	Second CES	Third CES	Sensitive to base case
Base Case	HPV 35–10	Pap smear 35–10	HPV 35–5	-
Life years gained (LYG)	Pap smear 35–10	Pap smear 35–5	Pap smear 30–5	Sensitive
Undiscounted QALY	HPV 35–10	HPV 35–5	Pap-smear 35–10	Sensitive
One-way sensitivity analysis of decreasing sensitivity of HPV DNA	Pap smear 35–10	Pap smear 35–5	Pap-smear 30–5	Sensitive
One-way sensitivity analysis of decreasing sensitivity of Pap smear	HPV 35–10	HPV 35–5	HPV 30–5	Sensitive
One-way sensitivity analysis of decreasing specificity of HPV DNA	Pap smear 35–10	HPV 35–10	Pap 35–5	Sensitive
One-way sensitivity analysis of decreasing specificity of Pap smear	HPV 35–10	Pap smear 35–10	HPV 30–10	Sensitive
One-way sensitivity analysis of increasing sensitivity of HPV DNA	HPV 35–10	HPV 35–5	HPV 30–10	Sensitive
One-way sensitivity analysis of increasing specificity of HPV DNA	HPV 35–10	Pap smear 35–10	HPV 35–5	Not sensitive
One-way sensitivity analysis of increasing specificity of Pap smear	Pap smear 35–10	Pap smear 35–5	Pap smear 30–5	Sensitive
One-way sensitivity analysis based on increasing the cost of HPV DNA	Pap smear 35–10	Pap smear 35–5	Pap smear 30–5	Sensitive
One-way sensitivity analysis based on increasing the cost of Pap smear	HPV 35–10	HPV 35–5	HPV 30–10	Sensitive

### Sensitivity Analysis

In one-way sensitivity analysis, our model was sensitive to the cost of the selected screening methods (i.e., Pap smear vs. HPV DNA testing) and test characteristics (Table 3). An increase or decrease in the sensitivity of screening methods affected the results and changed the ICERs. However, the model was not sensitive to the higher specificity of HPV DNA testing.

## Discussion

We performed a cost-effectiveness study and evaluated 11 cervical screening strategies using Pap smear and HPV DNA testing with different starting ages and testing intervals in Iran. Compared with a no-screening strategy, the most cost-effective strategy (ICER of $8,875 per QALY) was HPV DNA testing beginning at age 35 years with 10-year screening intervals. We showed that compared with a no-screening situation, an organized screening program may decrease the incidence of ICC from 974 to 483 women (26% to 63%), depending on the screening strategy.

To the best of our knowledge, this is the first economic evaluation for cervical screening in a Muslim country with low incidence of ICC. The results of this study could be generalized to other countries with the same risk profile. However, we faced some limitations. We lacked local data about the natural history of ICC, incidence rate of HPV in different age groups, and characteristics of HPV testing; we therefore used data from the international literature [45–50]. These types of data do not usually vary from country to country, and most economic studies rely on such data [45, 51, 52]. Although we used incidence rate of HPV infection based on reports from other countries, we eliminated potential bias through calibration and adjustment of the results with the existing prevalence rate of infection from Iran.

The starting age and intervals of cervical screening are important factors for its effectiveness and low cost. Different countries select screening strategies vis-à-vis these two factors. Although an earlier starting age and shorter intervals between screening lead to a higher number of tests during a woman’s lifetime, these may improve the effectiveness of a screening program. However, frequent screening would increase the burden and cost for the participant, health care providers, and governments. Therefore, it has always been challenging for governments to choose the best starting age and intervals for screening. HPV infection is a sexually transmitted disease and the prevalence of this infection is higher among women younger than 30 years of age [12]. However, women older than 30 years with persistent HPV infection are a group at high risk for ICC [53]. As we showed in this study, beginning screening at 35 years of age was more cost-effective than beginning at younger ages. This finding is supported by previous studies from other countries [17]. In practice, several countries including Finland, Korea, the Netherlands, and China begin screening at age 30 [54, 55] or 35 years [31, 34, 56]; screening starts at 25 years of age in the United Kingdom, France, Italy, and Portugal [55, 57], and age 21 in the United States and Canada [55].

Screening intervals also vary between countries. Although screening is performed every 3 years in the United States, New Zealand, and Norway, the intervals for screening is 5 years in many countries such as Denmark, Finland, and the Netherlands [55]. Many economic evaluation studies conducted in low- and middle-income countries have reported that 10- and 5-year intervals were more cost-effective than 3-year or shorter intervals [31, 34, 45, 56, 58]. Our study showed that expanding the screening interval to 10 years is more cost-effective than 5- or 3-year screening intervals. However, based on GDP per capita, 5-year intervals was also within the cost-effective range. In a systematic review, we found that the governments of most countries choose screening intervals conservatively, and national guidelines usually recommend 5-year intervals for cervical screening [17]. Therefore, although HPV DNA testing from age 35 years and repeated every 10 years was the most cost-effective strategy, 5-year intervals could be considered for cervical screening in Iran.

In line with a recent systematic review, we found that HPV DNA testing was a more cost-effective method compared with Pap smear for cervical screening and prevention of ICC [17]. This result may encourage policy makers to plan for implementation of HPV DNA testing in Iran and other low incidence populations. There are other advantages to shifting from conventional Pap smear to HPV DNA techniques [59–61]. HPV DNA testing is more acceptable and more convenient for both participants and providers [11, 62]. HPV DNA testing can be done using self-sampling methods and can thus improve the coverage of screening in target populations [62]. The use of HPV DNA testing for cervical screening is still evolving. Although most HPV testing strategies are combined with Pap smear triage, randomized trials have shown the superiority of HPV DNA testing alone compared with Pap smear screening [63]. In 2014, the US Food and Drug Administration (FDA) approved primary screening using HPV DNA testing [64], which has a simpler procedure and lower screening cost yet is as effective as other HPV DNA testing strategies [65]. Furthermore, using more sensitive methods such as cobas^®^ or Hybrid Capture 2 rather than PCR can improve the cost-effectiveness of HPV DNA testing for cervical screening [66].

## Conclusion

We performed a cost-effectiveness study and showed that organized cervical screening using HPV DNA testing, starting from age 35 and repeated every 10 years, was the most cost-effective strategy for Iran, a middle-income country with a low incidence rate of ICC. Our findings reflect that a more conservative approach was also cost-effective based on GDP per capita in Iran, with screening from age 35 with 5-year intervals. We suggest conducting a demonstration project in a limited geographical area of the country to evaluate the feasibility and cost of an organized screening program using HPV DNA testing. The results of such a study can provide empirical evidence for the effectiveness and cost-effectiveness of screening by HPV DNA testing, to help pave the way for a national program in the near future.
